# Essays on Infant Therapeutics

**Published:** 1849

**Authors:** 


### ARTICLE I.

*Essays on Infant Therapeutics*—to which are added Observations on Ergot, and an account of the origin of the use of Mercury in Inflammatory Complaints. By JOHN B. BECK, M. D., Prof. Mat. Med. and Med. Jurisprudence in the Colleges of Physicians and Surgeons of the University of the State of New York, &c., &c. pp. 117, 12mo. New York, W. E. Dean, printer and publisher, 1849.

This series of essays, comprising one on each of the subjects of the use of Opium, Emetics, Mercury, Blisters, and Bloodletting, in the young subject; which we have had the pleasure of laying before our readers in former numbers of the Journal, with the additional articles specified in the title, form the neatly got up little volume before us. As our readers are aware, it forms a valuable contribution to the literature of the medical profession. No praise of ours, however, could add to the well deserved reputation of these essays, as they are so extensively known and highly appreciated already. E.

